# Calcitriol Attenuates Doxorubicin-Induced Cardiac Dysfunction and Inhibits Endothelial-to-Mesenchymal Transition in Mice

**DOI:** 10.3390/cells8080865

**Published:** 2019-08-09

**Authors:** Tzu-Hsien Tsai, Cheng-Jei Lin, Chi-Ling Hang, Wei-Yu Chen

**Affiliations:** 1 Division of Cardiology, Department of Internal Medicine, Kaohsiung Chang Gung Memorial Hospital, Kaohsiung 83301, Taiwan; 2 Chang Gung University College of Medicine, Kaohsiung 83301, Taiwan; 3 Institute for Translational Research in Biomedicine, Kaohsiung Chang Gung Memorial Hospital, Kaohsiung 83301, Taiwan

**Keywords:** endothelial-to-mesenchymal transition, doxorubin, cardiac fibrosis, calcitriol

## Abstract

Doxorubicin (Dox) is an effective anti-neoplasm drug, but its cardiac toxicity limits its clinical use. Endothelial-to-mesenchymal transition (EndMT) has been found to be involved in the process of heart failure. It is unclear whether EndMT contributes to Dox-induced cardiomyopathy (DoIC). Calcitriol, an active form Vitamin D3, blocks the growth of cancer cells by inhibiting the Smad pathway. To investigate the effect of calcitriol via inhibiting EndMT in DoIC, C57BL/6 mice and endothelial-specific labeled mice were intraperitoneally administered Dox twice weekly for 4 weeks (32 mg/kg cumulative dose) and were subsequently treated with or without calcitriol for 12 weeks. Echocardiography revealed diastolic dysfunction at 13 weeks following the first Dox treatment, accompanied by increased myocardial fibrosis and up-regulated pro-fibrotic proteins. Calcitriol attenuated Dox-induced myocardial fibrosis, down-regulated pro-fibrotic proteins and improved diastolic function. Endothelial fate tracing revealed that EndMT-derived cells contributed to Dox-induced cardiac fibrosis. In vitro, human umbilical vein endothelial cells and mouse cardiac fibroblasts were treated with Transforming growth factor (TGF)-β with or without calcitriol. Morphological, immunofluorescence staining, and Western blot analyses revealed that TGF-β-induced EndMT and fibroblast-to-myofibroblast transition (FMT) were attenuated by calcitriol by the inhibition of the Smad2 pathway. Collectively, calcitriol attenuated DoIC through the inhibition of the EndMT and FMT processes.

## 1. Introduction

The mortality rates in patients with cancer have decreased over the last decades, but the numbers of patients suffering from the long-term side effects of antineoplastic drugs are increasing [1,2,3]. Doxorubicin (Dox) is an effective antineoplastic agent which is widely used for the treatment of several forms of adult and pediatric cancers including solid tumors, leukemia, lymphomas, and breast cancer [1,2,3,4,5,6,7,8]. The onset of doxorubicin-induced cardiotoxicity (DoIC) could be acute, occurring within 2–3 days [9,10,11], or be delayed until as long as 10–15 years after the end of chemotherapy [12,13,14]. The incidence of DoIC is around 11–18%, as reported by previous studies [11,13,15]. The use of Dox is limited by its cardiotoxicity. DoIC leads to congestive heart failure, which is a growing concern for cardiologists and oncologists. There is no effective treatment against the progression of cardiac remodeling and for the improvement of prognosis in patients with DoIC.

Endothelial-to-mesenchymal cell transition (EndMT) is a newly identified mechanism in the development of heart failure [16,17]. However, limited studies have focused on the role of EndMT in the development of DoIC. Calcitriol is an active form of vitamin D, which exerts its biological activity by binding to the vitamin D receptor to maintain endothelial function and by increasing the levels of endothelial nitric oxide synthase (eNOS) and vascular endothelial growth factor (VEGF) [18,19,20]. Epidemiological studies show that vitamin D3 supplementation is effective in the improvement of vascular function [21,22] and has a beneficial effect on the function of the left ventricle in patients with heart failure [23,24,25]. In addition, vitamin D has been shown to inhibit the migration and growth of cancer cells [26,27,28,29]. However, it is unknown if calcitriol is also able to attenuate the progression of cancer cells apart from its beneficial effect in the inhibition of DoIC. In the present study, we investigated the mechanism and beneficial effect of calcitriol in a preclinically-established mouse model of DoIC and revealed the potential implication of calcitriol for the attenuation of Dox-associated cardiac remodeling.

## 2. Materials and Methods

### 2.1. Animals

All animal experiments were approved by the Institutional Animal Care and Use Committee of Kaohsiung Chang Gung Memorial Hospital, Kaohsiung, Taiwan and carried out in accordance with the Guide for the Use and Care of Laboratory Animals. Wild-type C57BL/6 mice were purchased from NLAC, Taiwan. C57BL/6-Tg (Tek-RFP, cre/ERT2) 27Narl (RMRC13162, GEMMS NLAC, Taiwan) mice were crossed with C57BL/6J-Tg (UBC-DsRed-emGFP) 22Narl (RMRC13119, GEMMS NLAC, Taiwan) mice to obtain double transgenic mice (Tek-CreERT2/DRG). The genotypes of the mice were confirmed with PCR using specific primers: green fluorescence protein (GFP) forward—TGAACCGCATCGAGCTGAAGGG; GFP reverse—TCCAGCAGGACCATGTGATCGC; Cre forward—CTAAACATGCTTCATCGTCGGTC; Cre reverse—TCTGACCAGAGTCATCCTTAGCG.

### 2.2. Mouse Model of Doxorubicin-Induction Cardiomyopathy (DoIC)

**Figure 1:**
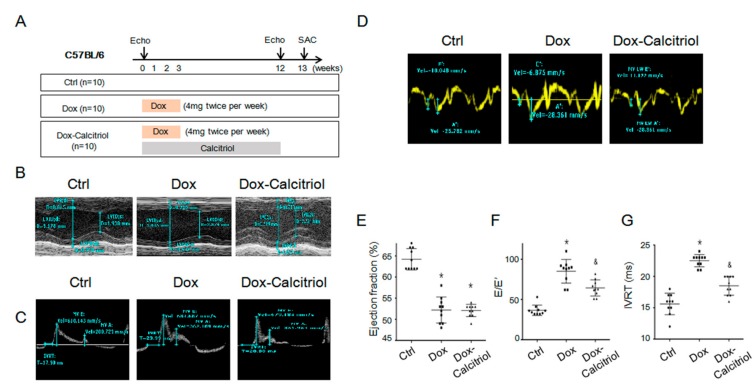
Calcitriol attenuated the doxorubicin-induced impairment of cardiac diastolic function. (**A**) The C57BL/6 mice were divided into 3 groups (n = 10, in each group). Group 1 (control group) received the same volume of saline injection, group 2 received doxorubicin (4 mg/kg twice weekly for 4 weeks; a cumulative dose of 32 mg/kg) and group 3 received doxorubicin (same dose as group 2) with calcitriol (150 ng/kg/day for 12 weeks). Echocardiography was performed on all the mice before the first dose of doxorubicin injection and 12 weeks after the first dose of doxorubicin injection. All the mice were sacrificed 13 weeks after the first dose of doxorubicin injection. (**B**) Representative M-mode of long-axis echocardiographic images for measurements of the IVSd, LVPWd, LVIDd, and LVIDs. (**C**) Representative sample of pulsed-wave Doppler revealing diastolic function: IVRT, peak velocity of the E wave and A wave. (**D**) Tissue Doppler measurements of septal mitral E’and A’. (**E**) Calculated ejection fraction of M-mode. (**F**) E/E’ ratio by pulse-wave Doppler and tissue Doppler. (**G**) Calculated IVRT by pulsed-wave Doppler. * *p* < 0.05, vs. Ctrl group. (n = 10 in each group), ^&^ *p* < 0.05, vs. Dox group. (n = 10 in each group). IVSd: interventricular septum thickness at mm end-diastole; LVPWd: left ventricular posterior wall thickness at end-diastole; LVIDd: left ventricular internal dimension at end-diastole; LVIDs: left ventricular internal dimension mm at end-systole, IVRT: iso-volemic relaxation time.

DoIC was induced in 10-week-old male C57BL/6 or double transgenic mice (Tek-CreERT2/DRG) mice (18–20 g) with 8 mg/kg doxorubicin (4 mg/kg intraperitoneally (i.p.), twice per week) for 4 consecutive weeks (a cumulative dose of 32 mg/kg) according to the modified method of a previous study [30]. The control group received a placebo at similar intervals, and the calcitriol treatment group received calcitriol (150 ng/kg/day). The dose and the protocol for this study were chosen based on the results of a previous study [31] and closeness to a clinical regimen. The detail experimental protocols are described in Figure 1A. Echocardiography was performed before doxorubicin injection and 12 weeks after the first dose of doxorubicin injection. The heart weight (HW) and the body weight (BW) were measured before the mice were sacrificed 13 weeks after the last doxorubicin injection. The mortality rate was about 30% in group 2 (doxorubicin-treated) and group 3 (doxorubicin and calcitriol-treated). A total of 10 mice that survived in groups 2 and 3 were included in this study.

Echocardiography was performed on animals under light anesthesia (1% isoflurane). Echocardiographic parameters such as heart rate (HR), parasternal long-axis, and short-axis views were recorded by a transthoracic echocardiography system (Vevo 2100, Visual Sonics, Toronto, ON, Canada) equipped with a high-frequency ultrasound probe. The end diastolic thickness of the intraventricular septum, left ventricular (LV) posterior wall thickness (LVPW), and end-systolic LV dimensions were measured on a parasternal short-axis M-mode. The fractional shortening (FS), ejection fraction (EF), LV volume, and LV mass index were calculated by Vevo 2100 software. The diastolic function was evaluated by a pulse wave Doppler spectral waveform and tissue Doppler spectral waveform. To measure the pulsed wave Doppler spectral waveforms, we measured the peak early and late-diastolic transmitral velocities (E and A waves) to obtain the E/A ratio and iso-volemic relaxation time (IVRT) by setting the sample volume at the tip of the mitral leaflets. To measure the tissue Doppler spectral waveforms at the septal mitral annulus, we measured E′ (early-diastolic myocardial relaxation velocity), A’, and calculated the E/E’.

### 2.3. Induction of GFP in Tek-Expressing Cells

The 8-week-old double transgenic mice (Tek-CreERT2/DRG) were treated with tamoxifen (20 mg/kg/day, i.p., Sigma-Aldrich) for 5 consecutive days to induce Cre-mediated deletion of the floxed DsRed allele. The recombined allele expressed GFP in the Tek-expressing cells. After a recovery period of at least 1 week after the first tamoxifen injection, the double transgenic mice (Tek-CreERT2/DRG) were subjected to DoIC induction. Tamoxifen was administered for 5 consecutive days before doxorubicin injection, and the mice were sacrificed 4 weeks and 13 weeks after the last doxorubicin injection. Approximately 98% of endothelial cells were labeled with GFP expression at 4 weeks after the last doxorubicin injection. However, the frequency of GFP+ endothelial cells diminished at 13 weeks, suggesting that a replenishment of endothelial cells by non-recombined endothelial cells occurs and that multiple tamoxifen injections may be required for optimal GFP-labeling in endothelial cells (data not shown). Therefore, to ensure GFP-labeled endothelial cells could be sustained during the experiment period, tamoxifen was administered for 5 consecutive days every four weeks to ensure the continuous expression of GFP in the endothelial cells (Supplementary Materials Figure S1A).

### 2.4. Cell Culture

Primary human umbilical vein endothelial cells (HUVECs) were purchased from Bioresource Collection and Research Center, Hsinchu, Taiwan. The HUVECs were cultured in M199 medium containing 5% fetal bovine serum (FBS), 1% endothelial cell growth factors (Millipore 02-102) and 1% penicillin/streptomycin in 5% CO_2_ at 37 °C. Only HUVECs at 3–6 passages were used for this experiment. The cells were cultured in 0.1% gelatin coating monolayer dishes. At approximately 80% confluence, the culture medium was changed to a serum-free solution for 24 h before the use of the cells in the experiments. The primary mouse cardiac fibroblasts were isolated from a pathogen-free neonate C57BL/6 mouse and cultured on pre-coated gelatin-based culture dishes as described in the previous study [31]. To examine the effect of TGF-β, the HUVECs or cardiac fibroblasts were treated with PBS, TGF-β1 (10 ng/mL) +10 ng TGF-β2 (10 ng/mL) or TGF-β1 (10 ng/mL) +10 ng TGF-β2 (10 ng/mL) with calcitriol (100 ng/mL) for 72 h according to the previous study [32]. Cell morphology, protein expression and gene expression were analyzed the EndMT markers using Western blot and immunofluorescent staining.

### 2.5. Histology and Immunofluorescent Staining

Paraffin-embedded sections of HUVECs cells were fixed in 4% paraformaldehyde for 20 min and then permeabilized in 0.03% Triton X-100 for 15 min. After being blocked in 5% bovine serum albumin for 30 min at room temperature, cells or slides were incubated with primary antibodies against CD31 (1:200; Ab28364, Abcam, Cambridge, UK), vimentin (1:200; Ab92547, Abcam, Cambridge, UK), GFP (1:200; SC-9996, Santa Cruz Biotechnology), (1:200; cell signaling:2535), troponin I (1:200; Ab47003, Abcam, Cambridge, UK) at 4 °C overnight, and then with a fluorescent dye-conjugated secondary antibody (Invitrogen). Nuclei were visualized with 4′6-diamidino-2-phenylindole·2HCl (Santa Cruz Biotechnology). Images were captured using an immunofluorescent microscope (Olympus BX5VCS3) or automated confocal laser-scanning microscope (Olympus FV10i). For cell immunofluorescence staining, HUVECs were fixed in 4% paraformaldehyde for 20 min and then permeabilized in 0.03% Triton X-100 for 15 min. After blocking in 5% bovine serum albumin for 30 min at room temperature, cells were incubated overnight at 4 °C with primary antibodies against smooth muscle 22α (SM22α) (1:200; Ab14106, Abcam, Cambridge, UK) and vascular endothelial (VE)-cadherin (1:100; sc-6458, Santa Cruz Biotechnology, Santa Cruz, CA, USA).

### 2.6. Reverse Transcription Quantitative Polymerase Chain Reaction (RT-qPCR)

Total RNA was extracted by using GENEzolTM TriRNA pure kit (Geneaid) according to the manufacturer’s directions. The cDNA was synthesized from 1 μg of total RNA and random hexamers by using the Transcriptor First Strand cDNA Synthesis kit (Roche). The forward and reverse primers were designed from sequences of different exons of the target gene to avoid amplifying genomic DNA. RT-qPCR analysis was carried out with QuantiNova TM SYBR Green PCR kit (QIAGEN) and StepOnePlus RT-qPCR system (Applied Biosystems), with 40 cycles of 95 °C × 5 sec and 60 °C × 1 min using 1 µL of diluted cDNA per 20 μL of reaction volume. All experiments were performed in triplicate. The primer sequences are presented in Supplementary Materials Table S1.

### 2.7. Western Blot Analysis

Cell lysates from cultured HUVECs, cardiac fibroblasts and heart tissue were extracted using Radioimmunoprecipitation assay buffer (RIPA) buffer (150 mM NaCl, 0.1% Sodium Dodecyl Sulfate (SDS), 0.5% sodium deoxycholate, 1 X protease inhibitor cocktail (Roche) and 50 mM Tris; pH 8.0). The total protein concentrations of cell extracts were determined by using a Bicinchoninic acid (BCA) protein assay kit (Pierce). SDS polyacrylamide gel and polyvinylidene fluoride (PVDF) membrane filter paper were used for immunoblotting. The washing buffer was 0.1% Tween 20 in Tris buffered saline (20 mM Tris and 150 mM NaCl; pH 7.4) and the blocking buffer was 5% skim milk (Bio-Rad) in washing buffer. The following proteins were analyzed: SM22-α (1:1000; Ab14106, Abcam, Cambridge, UK), CD31(1:200; Ab28364, Abcam, Cambridge, UK), fibronectin(1:200; Ab2413 Abcam, Cambridge, UK), alpha-smooth muscle actin (1:1000; Ab5694, Abcam, Cambridge, UK), sarcoplasmic/endoplasmic reticulum calcium ATPase (SERCA) (1:1000, GeneTex, GTX111808), total Smad2/3(1:1000, Cell signaling:8685), and phospholated-Smad2(1:1000, Cell signaling:3108).

### 2.8. Statistics

All data are presented as mean ± SEM or SD. Statistical analyses were performed using SPSS 18.0 (SPSS Inc, Chicago, IL, USA). Data were analyzed using Student’s t-test or one-way ANOVA, followed by a Bonferroni multiple-comparison post hoc test. A *p* value <0.05 indicated statistical significance.

## 3. Results

### 3.1. Calcitriol Attenuated Doxorubicin-Induced Impariment of Cardiac Diastolic Function

Our study focused on the late cardiotoxicity induced by doxorubicin (Dox), and experiments were modified from previous reports [30,33]. The C57BL/6 mice were divided into 3 groups (n = 10, in each group). Group 1 (control group) received the same volume of saline injection, group 2 received doxorubicin (4 mg/kg twice weekly for 4 weeks; a cumulative dose of 32 mg/kg) and group 3 received doxorubicin (same dose as group 2) with calcitriol (150 ng/kg/day for 12 weeks). Echocardiography was performed on all mice before the first dose of doxorubicin injection and 12 weeks after the first dose of doxorubicin injection. All the mice were sacrificed 13 weeks after the first dose of doxorubicin injection. The cardiac function of the mice was assessed by echocardiography 7 days prior to the first Dox injection (as the baseline) and 12 weeks after the first Dox injection (as the experiment endpoint) (Figure 1A–D and Table 1). There was no difference in the baseline cardiac functions between the groups before Dox injection (Table 1).

Echocardiaography by the M-mode of the parasternal long-axis revealed a thinner left ventricle septum (Figure 1B and Table 1) and a reduced ejection fraction of the left ventricle in the Dox-treated group and the Dox–calcitriol-treated group than those of the control group (Figure 1F). Additionally, calcitriol treatment did not alter Dox-reduced cardiac size and the ejection fraction (Figure 1F and Table 1). Pulsed-wave tissue Doppler revealed that Dox treatment induced a reduction in E/A ration (Figure 1D) and an increase in E/E’ (Figure 1F) and IVRT (Figure 1G), whereas calcitriol treatment reversed these phenomena, indicating an improvement of diastolic function by calcitriol in mice with DOX-induced cardiomyopathy (DoIC).

### 3.2. Calcitriol Reduced Dox-Induced Heart Failure Markers but did not Prevent Dox-Reduced Cardiac Sizes

**Figure 2:**
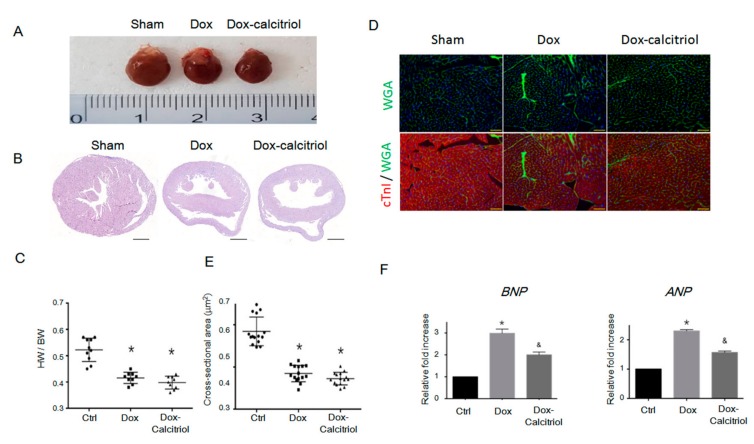
Effect of calcitriol on doxorubicin-induced cardiomyopathy. (**A**) Representative gross images of the whole heart. (**B**) Representative images of hematoxylin and eosin (H/E) staining of the ventricular sections. Scale bar: 1000 μm. (**C**) Quantification of the HW/BW ratio (n = 10 in each group). (**D**) Representative images of labeled WGA (green) and cardiac troponin I (red) staining of the ventricular sections. Scale bar: 20 μm. N = 3 in each group, 5 high power field (HPFs) per mouse, 15 HPFs total. (**E**) Quantification of the relative myocytes cross-sectional area (n = 3, 100 cells per field, 5 field in each heart). (**F**) Quantitative reverse transcription polymerase chain reaction (qRT-PCR) analyses of the mRNA levels of atrial Natriuretic Peptide (ANP) and brain natriuretic peptide (BNP). Data are normalized to the β-actin content (n = 3). Data are expressed as mean ± SEM, and n represents the number of animals. * *p* < 0.01, vs. Ctrl; ^&^ *p* < 0.05, vs. Dox. HW: heart weight; BW: body weight; WGA: wheat germ agglutinin.

To investigate the structural changes in the heart in DoIC, we analyzed the cardiac size, the heart weight (HW) to body weight (BW) ratio, and histological changes by hematoxylin and eosin (H&E) staining (Figure 2A–B). We found reduced heart sizes and lower HW/BW ratios in the Dox group and the Dox–calcitriol group than in the control group (Figure 2A–C). Calcitriol treatment, however, did not alter Dox-reduced heart sizes and HW/BW ratios (Figure 2C). We performed immunofluorescence (IF) staining to label cardiac troponin I (cTnI) and cell membranes by wheat germ agglutinin (WGA) for the evaluation of the cross-sectional area of cardiomyocytes between different groups (Figure 2D). Dox treatment reduced the cross-sectional area of cardiomyocytes, and calcitriol did not alter the cardiomyocyte sizes (Figure 2D–E). These results and those of the echocardiography demonstrated that Dox reduced the sizes of the heart and the cardiomyocytes, and these effects were not attenuated by calcitriol. However, qRT-PCR analysis revealed that calcitriol attenuated the Dox-induced elevation of the levels of heart failure markers atrial Natriuretic Peptide (ANP) and brain natriuretic peptide (BNP), suggesting that calcitriol modulates cardiac remodeling at the molecular level (Figure 2F).

### 3.3. Calcitriol Attenuated Dox-Induced Cardiac Fibrosis and Pro-Fibrotic Protein Expression

**Figure 3:**
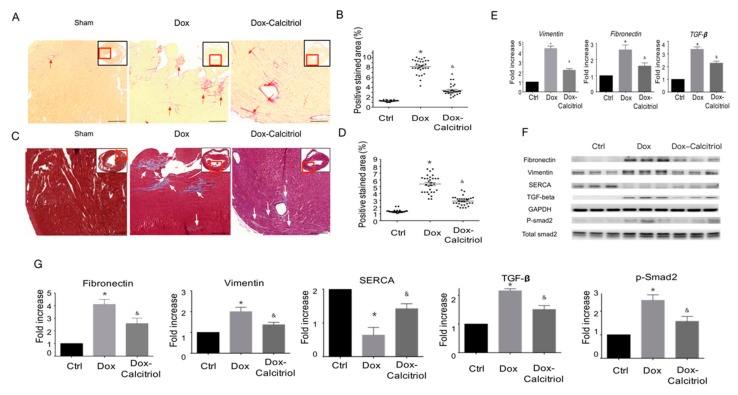
Calcitriol inhibits doxorubicin-induced cardiac remodeling. (**A**) Representative images of Picro-Sirius Red staining of the ventricular sections. The red arrowheads indicate the positive Picro-Sirius Red staining area. Scale bar: 50 μm. (**B**) Quantification of the relative fibrotic area (n = 3 in each group, 3 fields in each heart). (**C**) Representative images of Masson’s trichrome staining of the ventricular sections. The white arrowheads indicate the myocardial fibrosis area.(n = 3 in each group, 3 field in each heart; right). Scale bar: 50 μm. (**D**) Quantification of the relative fibrotic area by Masson’s trichrome staining (n = 3 in each group, 3 field in each heart). (**E**) qRT-PCR analyses of the mRNA expression of *Vimentin*, *Fibronectin*, and *TGF-β*. The data are normalized to the β-actin content (n = 3 in each group). Data are expressed as mean ± SEM, * *p* < 0.05, vs. Ctrl; & *p* < 0.05, vs. Dox. (**F**) Representative immunoblotting analyses of fibronectin, vimentin, sarcoplasmic/endoplasmic reticulum calcium ATPase (SERCA), TGF-β, and p-Smad2 in the heart tissue. (**G**) Quantification of the relative protein levels (n = 3). GAPDH was used as ian nternal control. Total-Smad2 as an internal control for p-Smad2. Data are expressed as mean ± SEM, * *p* < 0.05, vs. Ctrl; & *p* < 0.05, vs. Dox.

Our in vivo study found that calcitriol improved Dox-induced diastolic function impairment, without affecting Dox-induced heart sizes, suggesting that calcitriol likely has a protective effect at molecular levels to prevent Dox-induced pathological remodeling and fibrosis. To test this hypothesis, we next examined the Dox-induced fibrosis of the cardiac tissue sections by Picro-Sirius Red staining (Figure 3A–B) and Masson’s trichrome staining (Figure 3C–D). The areas with collagen deposition in the Dox-treated mice increased significantly as shown by Picro-Sirius Red and Masson’s trichome staining compared with the control group (Figure 3A–D). Treatment with calcitriol reduced fibrosis in Dox-treated mice by significantly reducing the level of collagen-rich tissue (Figure 3A–D). The qRT-PCR analysis was performed to investigate the effect of calcitriol on the expression of pro-fibrotic proteins associated with DoIC. We found that calcitriol significantly attenuated the Dox-induced up-regulation of pro-fibrotic gene expressions (vimentin, fibronectin, and TGF-β) (Figure 3E).

Western blot analysis also revealed the Dox-induced up-regulation of pro-fibrotic protein levels (vimentin, fibronectin, TGF-β, and p-Smad2), and the down-regulation of sarcoplasmic/endoplasmic reticulum calcium ATPase (SERCA), a marker for cardiac contractility function [34], whereas the alterations of those proteins by Dox were reversed in the Dox–calcitriol group, indicating that calcitriol inhibited Dox-induced pro-fibrotic responses (Figure 3F–G).

### 3.4. Calcitriol Attenuated Doxorubicin-Induced EndMT in Mouse Model

**Figure 4:**
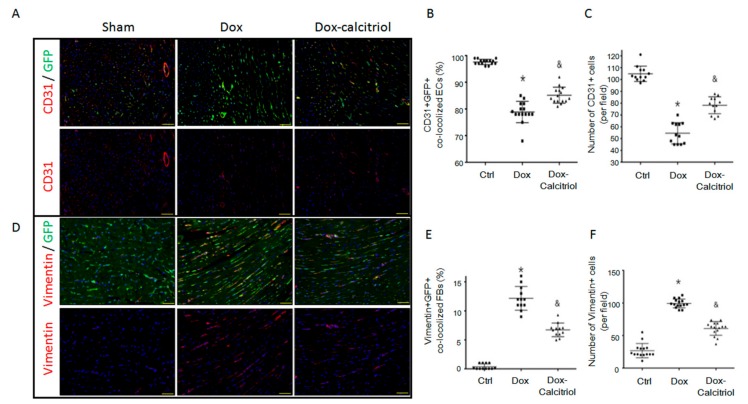
Calcitriol attenuated doxorubicin-induced endothelial-to-mesenchymal transition (EndMT) in the mouse model. (**A**) Representative photomicrographs of double immunofluorescence staining for the antibodies: CD31 (red) and GFP (green). (**B**) Quantification of CD31^+^/GFP^+^ cells per visual field in the Ctrl, Dox and Dox–calcitriol groups. (**C**) Representative photomicrographs of double immunofluorescence staining for the antibodies: CD31 (red). Quantification of CD31^+^ cells per visual field in the Ctrl, Dox, and Dox–calcitriol groups. (**D**) Representative photomicrographs of immunofluorescence staining for the antibodies: GFP (green) and vimentin (red). (**E**) Quantification of GFP^+^ and Vimentin^+^ cells per visual field in the Ctrl, Dox, and Dox–calcitriol groups. (**F**) Representative photomicrographs of double immunofluorescence staining for the antibodies: vimentin (red). Quantification of vimentin^+^ cells per visual field in the Ctrl, Dox and Dox–calcitriol groups. n = 3 mice per group, (5 high power field (HPFs) per mouse, 15 HPFs total). Scale bar: 200 μm; Data are expressed as mean ± SEM, and n represents the number of animals. * *p* < 0.05 vs. Ctrl, & *p* < 0.05 vs. Dox.

To further examine whether cells of endothelial origin contribute to the process of Dox-induced cardiac fibrosis via the endothelial-to-mesenchymal-transition (EndMT), we employed Tek-CreERT2;UBC-DsRed-emGFP (TekCreERT2/DRG) double transgenic mice. The TekCreERT2/DRG mice were generated by crossbreeding the TekCreERT2 mice with UBC-DsRed-emGFP reporter mice. To determine whether endothelial cells were specifically labelled, we injected the TekCreERT2/DRG mice with tamoxifen at a dose of 20 mg/kg/d for 5 days and analyzed the GFP expression in the heart tissues (Supplementary Materials Figure S1A and Figure 4A). After tamoxifen-induced Cre recombination, CD31+ endothelial cells were approximately 98% labeled by the cellular expression of GFP (Figure 4B), which confirmed the specificity of genetic labelling in endothelial cells of TekCreERT2/DRG mice. The double transgenic (TekCreERT2/DRG) mice had DoIC induced with or without calcitriol treatment and were sacrificed at 13 weeks after the first dose of Dox treatment. Dox treatment resulted in a reduced percentage of CD31+GFP+ co-localized cells (Figure 4B) as well as a reduced total of CD31+ vessels in the hearts, whereas calcitriol ameliorated Dox-induced endothelial loss (Figure 4C). Furthermore, co-staining with vimentin, a mesenchymal marker, with GFP revealed that Dox increased the frequency of Vimentin+GFP+ co-localized cells (Figure 4D–E), as well as causing an increased number of Vimentin+ cells in the hearts, whereas this was attenuated by calcitriol (Figure 4F). These together suggested that EndMT-derived mesenchymal cells contribute to Dox-induced cardiac fibrosis and that calcitriol could attenuate Dox-induced EndMT.

### 3.5. Calcitriol did not Inhibit Dox-Induced DNA Damage of Cardiac Myocytes and Endothelial Cells

We next investigated whether Dox-mediated DNA damage contributes to Dox-induced EndMT. By using immunofluorescent staining to detect the expression of γ-H2AX, a marker for DNA damage, in cTnI+ cardiomyocytes (Supplementary Materials Figure S1B), we found that ~7% of GFP+ cells (including endothelial cells and EndMT-derived mesenchymal cells) co-localized with γ-H2AX signaling in the nuclei in the Dox group (Supplementary Materials Figure S1C). About 25% of the cardiomyocytes co-localized with γ-H2AX signaling in the nuclei in the Dox-treated hearts (Supplementary Materials Figure S1D–E). Calcitriol, however, did not alter the frequency of γ-H2AX+ cells in cTnI+ cardiomyocytes nor in GFP+ cells in the heart tissue, suggesting that calcitriol has a minimal effect on Dox-induced DNA damage of cardiomyocytes and endothelial cells. We also performed TUNEL(Terminal deoxynucleotidyl transferase (TdT) dUTP nick end labeling) staining to evaluate the degree of cardiomyocyte apoptosis in our study. However, in line with the results of IF staining for the DNA damage marker γ-H2AX, we did not observe significant differences in the number of TUNEL+ cardiomyocytes between the Dox and Dox-calcitriol groups (Supplementary Materials Figure S1F–G).

### 3.6. Calcitriol Inhibited Dox-Induced p-Smad2 Activation in Endothelial Cells In Vivo

**Figure 5:**
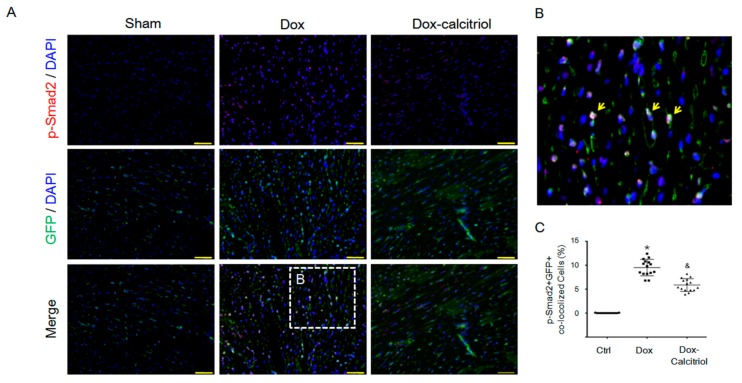
Calcitriol-attenuated doxorubicin-induced Smad2 activation in vivo. (**A**) Representative photomicrographs of double immunofluorescence staining antibodies with p-Smad2 (red) and GFP (green) in different groups. (**B**) Enlarged area from (**A**, white box). The yellow arrows indicated p-Smad2+ co-localized in GFP^+^ cells. (**C**) Quantification of p-Smad2+/GFP^+^ cells per visual field in the Ctrl, Dox, and Dox–calcitriol groups. N = 3 mice per group (5 high power field (HPFs) per mouse, 15 HPFs total). Scale bar: 200 μm. Experiments were conducted in triplicate. Data are expressed as mean ± SEM, and n represents the number of animals. * *p* < 0.05 vs. Ctrl, & *p* < 0.05 vs. Dox.

The TGF-β–Smad pathway has been shown to be the major regulator for EndMT [35]. We next performed IF staining to detect the expression of p-Smad2, a downstream signaling molecule activated by TGF-β, in the hearts (Figure 5A–B). Dox treatment increased the percentage of p-Smad2+/GFP+ cells, whereas calcitriol co-treatment significantly reduced the p-Smad2+/GFP+ cells (Figure 5C), suggesting that calcitriol treatment attenuated Dox-induced EndMT by the inhibition of the Smad2 pathway.

### 3.7. Calcitriol Attenuated the EndMT and FMT Process by the Suppression of TGF-β1 in the In Vitro Model

**Figure 6:**
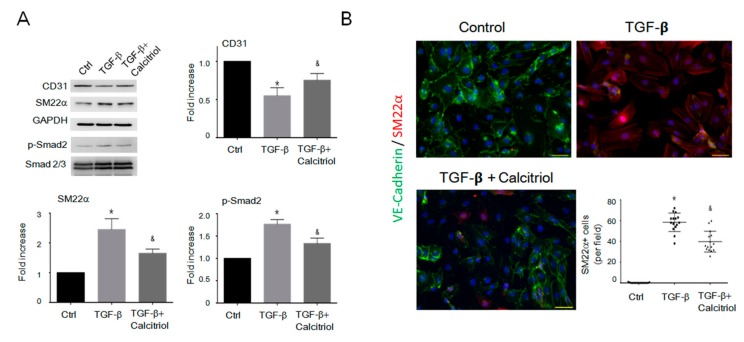
Calcitriol-attenuated TGF-β-induced EndMT in vitro. (**A**) Representative immunoblotting analyses of CD31, smooth muscle 22alpha, and p-Smad2 in the human umbilical vein cells (HUVECs). Quantification of the relative protein levels (n = 3). GAPDH was used as an internal control, and total Smad2 as an internal control for p-Smad2. Data are expressed as mean ± SEM. * *p* < 0.05 vs. Ctrl, & *p* < 0.05 vs. TGF-β. Experiments were conducted in triplicate. (**B**) Representative photomicrographs of double immunofluorescence staining antibodies with SM22α (red) and VE-cadherin (green). Quantification of vimentin^+^ cells per visual field in the Ctrl, TGF-β.only, and TGF-β–calcitriol groups. Experiments were conducted in triplicate (5 high power fields (HPFs) per mouse, 15 HPFs total). Scale bar: 200 μm * *p* < 0.05 vs. Ctrl, & *p* < 0.05 vs. TGF-β.

Although we found that calcitriol effectively attenuated DoIC and EndMT in mice, it is unclear if calcitriol is also an effective inhibitor of EndMT in vitro. HUVECs and mouse cardiac fibroblasts were used to test this hypothesis. Immunoblotting analysis confirmed that TGF-β reduced the expression of the endothelial cell marker (CD31) and increased the expression of the mesenchymal markers (vimentin and SM22α) as well as the p-Smad2 in HUVECs (Figure 6A). Treatment with calcitriol blocked the TGF-β-induced down-regulation of CD31 and the up-regulation of mesenchymal cells markers (vimentin and SM22α) through the inhibition of p-Smad2 (Figure 6A).

IF staining revealed that the incubation of HUVECs with TGF-β for 48 h increased the percentage of VE-cadherin^+^/SM22α^+^ cells, indicating that TGF-β-triggered EndMT in vitro (Figure 6B). Calcitriol, however, reduced the percentage of SM22α^+^ cells among total VE-cadherin^+^ endothelial cells when compared with the TGF-β-treated group (Figure 6B). Together, calcitriol inhibited the TGF-β-induced loss of endothelial phenotypes and prevented EndMT progression in vitro.

**Figure 7:**
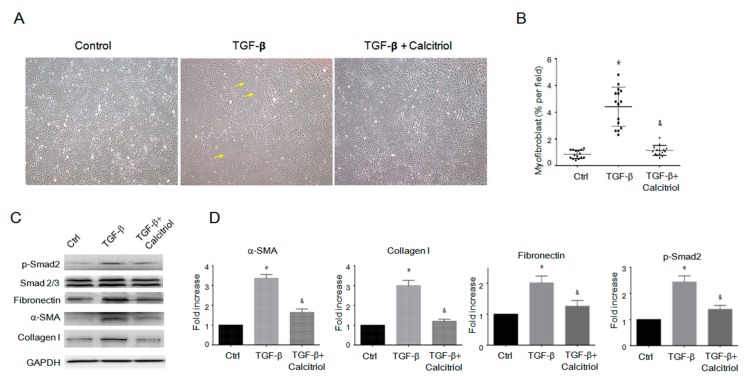
Calcitriol-attenuated TGF-β-induced fibroblast-to-myofibroblast transition (FMT) in vitro. (**A**) Representative photomicrographs of the myofibroblast. (**B**) Quantification of myofibroblast cells per visual field in the Ctrl, TGF-β only, and TGF-β–calcitriol groups. Experiments were conducted in triplicate (5 high power fields (HPFs) per mouse, 15 HPFs total). * *p* < 0.05 vs. Ctrl, & *p* < 0.05 vs. TGF-β. (**C**) Representative immunoblotting analyses of fibronectin, alpha-SMA, collagen I, and p-Smad2 in the cardiac fibroblasts. (**D**) Quantification of the relative protein levels (n = 3). GAPDH was used as an internal control. Total Smad2 was used as an internal control for p-Smad2. Data are expressed as mean ± SEM, and n represents the number of animals. * *p* < 0.05 vs. Ctrl, & *p* < 0.05 vs. TGF-β. Experiments were conducted in triplicate.

Finally, we tested if calcitriol would inhibit fibroblast-to-myofibroblast transition (FMT) from the inhibition of the activation of endogenous cardiac fibroblasts. We isolated and cultured cardiac fibroblasts with TGF-β with our without calcitriol. Morphological analysis revealed that calcitriol inhibited the TGF-β-increased number of large, spindle-shaped myofibroblasts (Figure 7A–B). Immunoblotting analyses revealed that the pro-fibrotic protein expressions (p-Smad2, fibronectin, α-smooth muscle actin (SMA), and collagen I) were upregulated by TGF-β, whereas calcitriol effectively reduced TGF-β-induced pro-fibrotic responses (Figure 7C–D). Taken together, our results demonstrated that calcitriol not only inhibited EndMT in endothelial cells but also FMT in the cardiac fibroblasts.

## 4. Discussion

**Figure 8:**
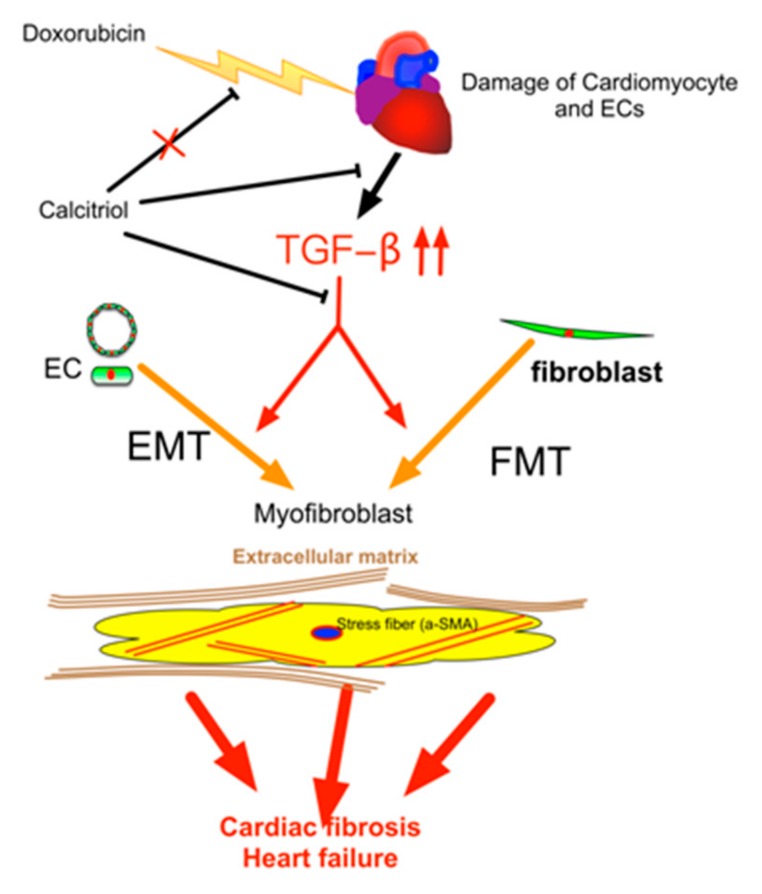
Schematic representation of the mechanism of action of calcitriol in the protection against DoIC-associated cardiac remodeling. Doxorubicin induced damage to cardiomyocytes and endothelial cells and caused subsequent tissue remodeling by the augmentation of the TGF-β pathway. Calcitriol attenuated Doxorubicin-caused cardiac remodeling by the attenuation of TGF-β-mediated EndMT and FMT.

Our study demonstrated that (1) calcitriol protected the mice against Dox-caused diastolic dysfunction and cardiac fibrosis; (2) the cells of endothelial origin were involved in DoIC-associated cardiac fibrosis and remodeling; (3) calcitriol attenuated Dox-induced EndMT and ECM production by the inhibition of the TGF-β–Smad2 pathway in the hearts of the DoIC mice; and (4) calcitriol attenuated TGF-β-induced EndMT and FMT by the inhibition of the Smad2 pathway in vitro. These results collectively suggest the beneficial effects of calcitriol in the protection against cardiac remodeling through the inhibition of EndMT and FMT in a DoIC mouse model (Figure 8).

Myocardial fibrosis with an abundant extracellular matrix (ECM) is the cause of diastolic dysfunction [36,37,38,39,40]. Clinical pathological studies reported the presence of myocardial fibrosis in patients with DoIC [41,42]. Diastolic dysfunction is also seen in cancer patients after doxorubicin therapy [43,44]. Our results and previous reports demonstrated late cardiac remodeling with cardiac fibrosis following Dox treatment [30]. The cumulative doxorubicin dose of 32 mg/kg (equivalent to ~96 mg/m^2^ in humans) was designed to mimic clinical treatment at low Dox doses (30–90 mg/m^2^) [45]. This dosage caused a slight decrease in systolic function but a significant decrease in diastolic dysfunction at 12-week follow-up [30]. We further showed that calcitriol treatment effectively attenuated cardiac fibrosis and inhibited the expression of pro-fibrotic proteins, which improved DoIC-associated cardiac remodeling. These findings may explain, at least in part, why calcitriol improved diastolic function. Interestingly, our study, in line with previous studies, showed that Dox caused cardiac atrophy and a reduction in the sizes of cardiac myocytes [46,47], whereas calcitriol treatment did not attenuate this process. The underling molecular mechanisms between Dox-caused cardiac atrophy and diastolic dysfunction, however, remain unclear and require further investigation.

EndMT has been shown to participate in the heart failure mice models induced by pressure overload and myocardial infarction [48], whereas the role of EndMT in DoIC is not well understood. Dox has been shown to cause damage to endothelial cells via increasing oxidative stress, which enhances drug permeability and promotes adverse effects for the cardiac myocytes [49]. IF staining for γ-H2AX with GFP or cTnI in the endothelial fate-mapped mice demonstrated that Dox not only causes damage to the cardiomyocytes but also the cardiac micro-vascular endothelial cells. Although previous studies have demonstrated that Dox directly causes the damage of endothelial cells in vitro [49,50], our study provided evidence that Dox causes the damage of endothelial cells in a mouse DoIC model.

The TGF-β–Smad axis played a critical role in EndMT [35]. Vitamin D was shown to inhibit TGF-β-induced fibrosis and myofibroblast formation [51,52,53,54]. Our result demonstrated that p-Smad2 was significantly decreased by calcitriol in the TGF-β-induced EndMT in HUVECs. In vivo, we also observed that calcitriol reduced the percentage of Smad2+/GFP+ cells in the doxorubicin-treated double transgenic mice with down-regulated fibronectin, vimentin and α-SMA. Additionally, our results also demonstrated that calcitriol inhibited the FMT and ECM production in cardiac fibroblasts in vitro. These together suggested that calcitriol ameliorated ECM production through the suppression of TGF-β–Smad-mediated EndMT and FMT. However, our results did not exclude the possibility that other vitamin D receptor agonists also have inhibitory effects on EndMT or FMT. Recent studies also found that other Vitamin D receptor agonists inhibit the process of tissue fibrosis. Interesting, vitamin D-deficient mice also attenuated the carbon tetrachloride-induced liver fibrosis. These findings demonstrated that the activation of vitamin D receptor had an effect on inhibiting the fibrosis process [51,53,54,55].

In our in vivo study, we analyzed the protein levels of TGF-β1 and found them to be upregulated in the Dox-treated heart while downregulated by calcitriol treatment. Therefore, the upregulation of tissue TGF-β1 levels is likely attributed to the induction of EndMT and FMT in the myocardium microenvironment. Endothelial cells have been shown to produce TGF-β [56,57,58]. Indeed, we did not analyze the production of TGF-β by endothelial cells, as other cell types might cause the production of TGF-β following Dox insults. Nonetheless, our in vitro result suggested that calcitriol inhibited EndMT in TGF-β-treated endothelial cells and TGF-β-treated fibroblasts, indicating that calcitriol inhibited TGF-β-related fibrotic responses in both cell types.

In clinical practice, calcitriol may not only inhibit neoplasm progression but also prevent doxorubicin-induced heart failure. Hence, the use of calcitriol should be encouraged in cancer patients undergoing doxorubicin therapy.

Our study had several limitations. Firstly, this study did not investigate the optimal dosage of calcitriol for maximizing the cardiac protective effects in the DoIC model. However, previous studies have demonstrated that a relatively high dose of calcitriol offered adequate protective effects in mouse models of polycystic ovary syndrome [59]. A recent study demonstrated that calcitriol (100 ng/kg/day) effectively increased angiogenic myeloid cells to improve vascular regeneration [60]. The dosage of calcitriol used in the present study (150 ng/kg/day) was the same as that of a previous study [59]. Secondly, the DoIC mice used in the present study were 8–12 weeks old and they were treated with calcitriol for 12 weeks. However, our study design did not properly reflect a clinical situation. Therefore, the results of this study should be carefully extrapolated to the clinical setting. Thirdly, we observed that the attenuation of EndMT was associated with the inhibition of the Smad2 pathway in this study; however, the precise signaling pathway(s) through which calcitriol exerted its therapeutic effects were not elucidated. Fourth, the effects of calcitriol on systemic parameters such as inflammatory cytokines and acute cardiac injury marker cardiac troponin I (cTnI) were not investigated in our study. Previous reports claimed that calcitriol reduced the levels of systemic inflammatory parameters. Inflammatory cytokines such as interleukin (IL)-1β also triggered EndMT; [61,62]; therefore, we do not exclude the possibility that calcitriol attenuated EndMT by inhibiting the systemic inflammation process in vivo [63,64].

In conclusion, our study revealed that EndMT is one of the mechanisms associated with the late-phase cardiac fibrosis in DoIC. Calcitriol is effective in the attenuation of Dox-induced cardiac fibrosis and improvement of diastolic function. The mechanism of action of calcitriol on DoIC-related cardiac dysfunction occurs partially through the inhibition of TGF-β–Smad2-mediated EndMT and FMT.

## Figures and Tables

**Table 1:** Echocardiography analysis for the cardiac functions.

	Ctrl	Dox	Dox–Calcitriol
Baseline			
LVSd (mm)	0.78 ± 0.13	0.79 ± 0.08	0.78 ± 0.11
LVPWd (mm)	0.63 ± 0.11	0.67 ± 0.15	0.68± 0.13
LVIDd (mm)	2.933 ± 0.31	2.98 ± 0.17	2.89 ± 0.16
LVIDs (mm)	1.938 ± 0.13	2.13 ± 0.18	2.13 ± 0.17
LV mass index	51.16 ± 22	53.1 ± 17	52.6 ± 23
EF (%)	66.7 ± 8.1	67.2± 7.2	66.7 ± 7.6
FS	35.9 ± 6.3	37.6 ± 5.4	36.0 ± 7.9
E/E′	46 ± 7.8	48 ± 6.2	45 ± 8.7
IVRT	15.3± 1.2	16.2 ± 1.8	15.7 ± 1.5
12 weeks later			
LVSd (mm)	0.82 ± 0.15	0.74 ± 0.16 *	0.72 ± 0.03 *
LVPWd (mm)	0.76 ± 0.16	0.62 ± 0.17 *	0.61 ± 0.06 *
LVIDd (mm)	3.3 ± 0.34	3.87 ± 0.13 *	3.78 ± 0.21 *
LVIDs (mm)	2.13 ± 0.12	2.88 ± 0.14 *	2.75 ± 0.18 *
LV mass index	82 ± 19	43 ± 16 *	45 ± 22 *
EF (%)	64 ± 8.1	51 ± 7.2 *	52 ± 5.3 *
FS	35.3 ± 7.2	25.2 ± 6.4 *	26.3 ± 6.2 *
E/E′	47 ± 3.8	87 ± 9.6 *	67 ± 12.8 ^&^
IVRT	15.7± 1.4	23.5 ± 1.7 *	19.6 ± 1.8 ^&^

IVSd, diastolic interventricular septum diameter; LVIDd, diastolic le ventricular internal diameter; LVIDs, systolic left ventricular internal diameter; LVPWd, diastolic left ventricular posterior wall diameter, EF: ejection fraction, FS: fractional shortening, E = early diastolic flow velocity, E′ = mitral annulus early diastolic velocity; IVRT: iso-volumic relaxation time; Data are expressed as mean ± SEM. * *p* < 0.05 vs. Ctrl and Dox–calcitriol groups and; ^&^ *p* < 0.05 vs. Dox group. Control: control group; Dox: Doxorubicin treated group, Dox–calcitriol: Doxorubicin with calcitriol treated group.

## Supplementary Materials

Supplementary Materials can be found at https://www.mdpi.com/2073-4409/8/8/865/s1.

## Abbreviations

cTnI	Cardiac troponin I
Dox	Doxorubicin
DoIC	Dox-induced cardiomyopathy
ECM	Extracellular matix
EndMT	Endothelial-to-mesenchymal transition
eNOS	endothelial nitric oxide synthase
FMT	Fibroblast-to-myofibroblast transition
HW/BW	Heart weight to body weight ratio
H&E	Hematoxylin and eosin
HUVECs	Human umbilical vein endothelial cells
IF	Immunofluorescence
IVSd	Interventricular septum thickness at mm end-diastole
IVRT	Iso-volemic relaxation time
LVPWd	Left ventricular posterior wall thickness at end-diastole
LVIDd	Left ventricular internal dimension at end-diastole
LVIDs	Left ventricular internal dimension mm at end-systole
qRT-PCR	Quantitative reverse transcrition polymerase chain reaction
SERCA	Sarcoplasmic/endoplasmic reticulum calcium ATPase
TekCreERT2/DRG	Tek-CreERT2;UBC-DsRed-emGFP doulble transgenic mice
VEGF	Vascular endothelial growth factor
WGA	Wheat germ agglutinin
