# Supplementary material for: Calcitriol Attenuates Doxorubicin-Induced Cardiac Dysfunction and Inhibits Endothelial-to-Mesenchymal Transition in Mice
Source: Cells. 2019 Aug 9;8(8):865. doi: 10.3390/cells8080865 (PMC6721693; doi:10.3390/cells8080865)
Supplement: Supplementary file 1 [file cells-08-00865-s001.pdf]

Supplemental Table S1. Primers used in this study

Gene name	Primer sequences
ANP	F: 5′- TGGGTCTTGTTAGGGCTCAAACCT -3′
	R:5′- TGAAACTCAAGGGACACCCATCGT -3′
BNP	F: 5′-CTGCTGGAGCTGATAAGAGA-3′
	R: 5′-TGCCCAAAGCAGCTTGAGAT-3′
Vimentin	F: 5′-GCAAAGATTCCACTTTGCGT-3′
	R: 5′-GAAATTGCAGGAGGAGATGC-3′
Fibronectin	F: 5′- ACCGAAGCCGGGAAGAGCAA -3′
	R: 5′- GGTCCGTTCCCACTGCTGATTTATC-3′
TGF-beta1	F: 5′- CGGAAGCGCATCGAAGCCATCC -3′
	R: 5′- GCAAGCGCAGCTCTGCACGG-3′
Beta-actin	F: 5′-CCAACCGCGAGAAGATGA-3′
	R: 5′-CCAGAGGCGTACAGGGATAG-3′

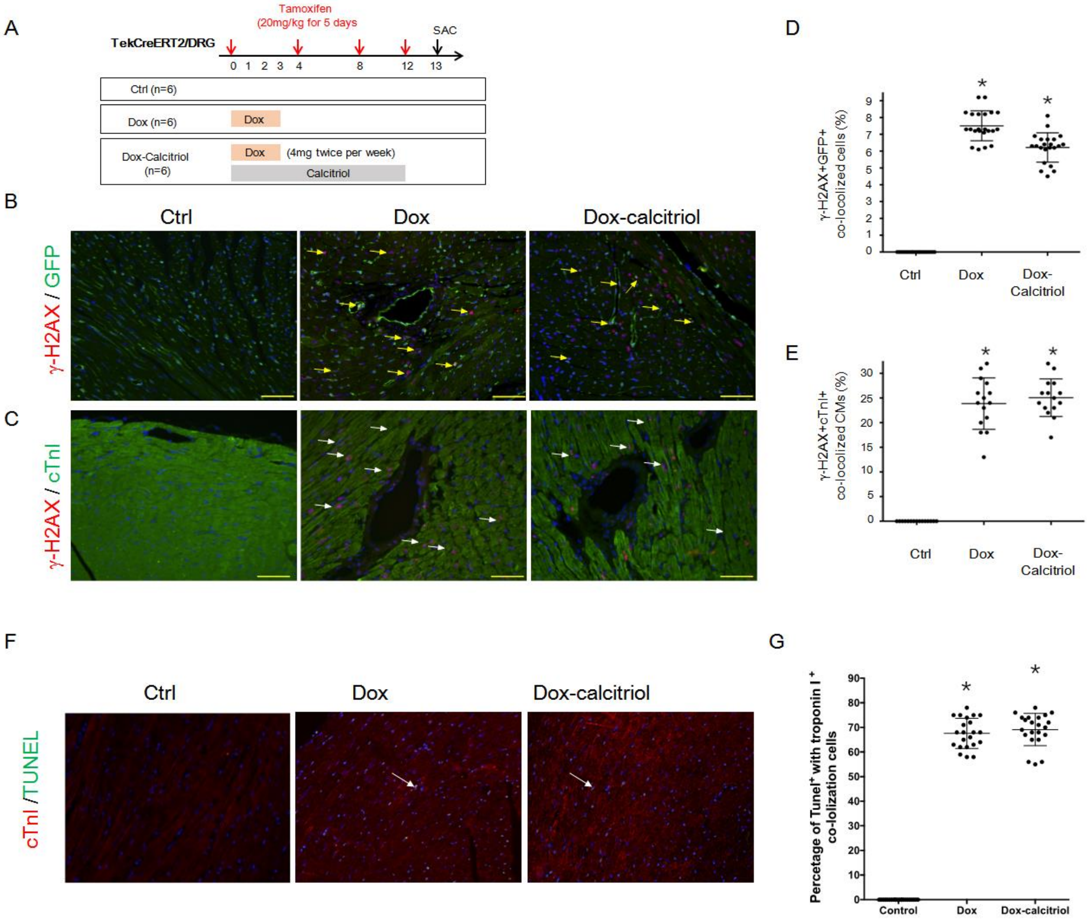

Supplemental Figure S1. Calcitriol did not alter doxorubicin-induced DNA damage in endothelial cells and cardiac myocytes. (A) Schematic protocol for Dox-induced cardiomyocytes in double transgenic mice. The TekCreERT2/DRG mice were divided into 3 groups (n=6, in each group). Group 1 (sham control) received same volume of saline injection, group 2 received doxorubicin (4 mg/kg twice weekly for 4 weeks; cumulative dose-32 mg/kg) and group 3 received doxorubicin (same dose as group 2) with calcitriol (150 ng/kg/day for 12 weeks). Before the doxorubicin injection, the mice were administered tamoxifen (20 mg/kg) for 5 consecutive days monthly. The mice were sacrificed at 4 weeks (n=3 in each group) and 13 weeks (n=3 in each group) after the first dose of doxorubicin injection. **(B)** Representative images of double immunofluorescence staining for γ-H2AX (red) and GFP (green). The yellow arrow heads indicate positive γ-H2AX and GFP co-localized cells. Data are expressed as mean ± SEM, and n represents the number of animals. * $p < 0.05$, $^{\$}$ $p < 0.01$ (n = 3, in each group), [5 high power field (HPFs) per mouse, 15 HPFs total]. Scale bar: 200 mm. (Upper panel, **(C)** Representative images of double immunofluorescence staining for γ-H2AX (red) and cardiac Troponin I (cTnI) (green). The white arrow heads indicate positive γ-H2AX and Troponin I co-localized cells. Scale bar: 200 mm. **(D)** Quantification of the percentage of γ-H2AX+GFP+ co-localized cells. Data are expressed as mean ± SEM, and n represents the number of animals. * P < 0.05 (n = 3 in each group), [5 high power field (HPFs) per mouse, 15 HPFs total]. **(E)** Quantification of the percentage of γ-H2AX+GFP+ co-localized cells. (F) Representative images of double immunofluorescence staining for TUNEL (green) and cardiac Troponin I (cTnI) (red). The white arrow heads indicate positive TUNEL and Troponin I co-localized cells. (G) Quantification of the percentage of TUNEL and Troponin I co-localized cells. Data are expressed as mean ± SEM, and n represents the number of animals. * P < 0.05 (n = 3 in each group), [5 high power field (HPFs) per mouse, 15 HPFs total].
